# Authors’ response – Occupation and SARS-CoV-2 infection risk among workers during the first pandemic wave in Germany: potential for bias

**DOI:** 10.5271/sjweh.4061

**Published:** 2022-10-01

**Authors:** Marvin Reuter, Mariann Rigó, Maren Formazin, Falk Liebers, Ute Latza, Stefanie Castell, Karl-Heinz Jöckel, Karin Halina Greiser, Karin B Michels, Gérard Krause, Stefan Albrecht, Ilter Öztürk, Oliver Kuss, Klaus Berger, Benedikt MJ Lampl, Michael Leitzmann, Hajo Zeeb, Karla Romero Starke, Sabine Schipf, Claudia Meinke-Franze, Wolfgang Ahrens, Andreas Seidler, Bianca Klee, Tobias Pischon, Andreas Deckert, Börge Schmidt, Rafael Mikolajczyk, André Karch, Barbara Bohn, Hermann Brenner, Bernd Holleczek, Nico Dragano

**Affiliations:** 1 Institute of Medical Sociology, Centre for Health and Society, Medical Faculty and University Hospital, University of Düsseldorf, Dusseldorf, Germany; 2 Federal Institute for Occupational Safety and Health (BAuA), Berlin, Germany; 3 Helmholtz Centre for Infection Research, Braunschweig, Germany; 4 Institute for Medical Informatics, Biometry and Epidemiology (IMIBE), University Hospital Essen, Germany; 5 German Cancer Research Centre (DKFZ) Heidelberg, Div. of Cancer Epidemiology, Heidelberg, Germany; 6 Institute for Prevention and Cancer Epidemiology, Faculty of Medicine and Medical Center, University of Freiburg, Freiburg, Germany; 7 Institute for Biometrics and Epidemiology, German Diabetes Center, Leibniz Center for Diabetes Research at Heinrich-Heine-University ­Düsseldorf, Düsseldorf, Germany; 8 Institute of Epidemiology and Social Medicine, University of Münster, Münster, Germany; 9 Regensburg Department of Public Health, Germany; 10 Department of Epidemiology and Preventive Medicine, University of Regensburg, Germany; 11 Leibniz Institute for Prevention Research and Epidemiology - BIPS, Bremen, Germany; 12 Institute and Policlinic for Occupational and Social Medicine, Faculty of Medicine, Technische Universität Dresden, Germany; 13 Institute for Community Medicine, University Medicine Greifswald, Greifswald, Germany; 14 Institute for Medical Epidemiology, Biometrics and Informatics, Interdisciplinary Center for Health Sciences, Medical Faculty of the Martin Luther University Halle-Wittenberg, Halle (Saale), Germany; 15 Max Delbrück Center for Molecular Medicine in the Helmholtz Association (MDC), Molecular Epidemiology Research Group, Germany; 16 Heidelberg Institute of Global Health, Heidelberg University, Heidelberg, Germany; 17 NAKO e.V. Heidelberg, Germany; 18 Division of Clinical Epidemiology and Aging Research, German Cancer Research Center (DKFZ), Heidelberg, Germany; 19 Krebsregister Saarland, Saarbrücken, Germany; 20 Institute for Infectious Disease Epidemiology, TWINCORE, Hannover, Germany; 21 German Center for Infection Research (DZIF), Braunschweig, Germany; 22 Robert Koch Institute, Department for Epidemiology and Health Monitoring, Germany

We thank van Tongeren et al for responding to our study on occupational disparities in SARS-CoV-2 infection risks during the first pandemic wave in Germany (1). The authors address the potential for bias resulting from differential testing between occupational groups and propose an alternative analytical strategy for dealing with selective testing. In the following, we want to discuss two aspects of this issue, namely (i) the extent and reasons of differential testing in our cohort and (ii) the advantages and disadvantages of different analytical approaches to study risk factors for SARS-CoV-2 infection.

Our study relied on nationwide prospective cohort data including more than 100 000 workers in order to compare the incidence of infections between different occupations and occupational status positions. We found elevated infection risks in personal services and business administration, in essential occupations (including health care) and among people in higher occupational status positions (ie, managers and highly skilled workers) during the first pandemic wave in Germany (2). Van Tongeren’s et al main concern is that the correlations found could be affected by a systematic bias because people in healthcare professions get tested more often than employees in other professions. A second argument is that better-off people could be more likely to use testing as they are less affected by direct costs (prices for testing) and the economic hardship associated with a positive test result (eg, loss of earnings in the event of sick leave).

We share the authors’ view that differential testing must be considered when analysing and interpreting the data. Thus, in our study, we examined the proportion of tests conducted in each occupational group as part of the sensitivity analyses (see supplementary figure S1, accessible at www.sjweh.fi/article/4037). As expected, testing proportions were exceptionally high in medical occupations (due to employer requirements). However, we did not observe systematic differences among non-medical occupations or when categorising by skill-level or managerial responsibility. This might be explained by several reasons. First, SARS-CoV-2 testing was free of charge during the first pandemic wave in Germany, but reporting a risk contact or having symptoms was a necessary condition for testing^1^. The newspaper article cited by van Tongeren et al is misleading as it refers to a calendar date after our study period. Second, different motivation for testing due to economic hardship in case of a positive test result is an unlikely explanation, because Germany has a universal healthcare system, including paid sick leave and sickness benefits for all workers (3). Self-employed people carry greater financial risks in case of sickness. We therefore included self-employment in the multivariable analyses to address this potential source of bias.

While the observed inverse social gradient may be surprising, it actually matches with findings of ecological studies from Germany (4, 5), the United States (6, 7) as well as Spain, Portugal, Sweden, The Netherlands, Israel, and Hong Kong (8), all of which observed higher infection rates in wealthier neighbourhoods during the initial outbreak phase of the pandemic. One possible explanation is the higher mobility of managers and better educated workers, who are more likely to participate in meetings and engage in business travel and holiday trips like skiing. Given the increasing number of studies providing evidence for this hypothesis, we conclude that the inverse social gradient in our study likely reflects different exposure probabilities and is not a result of systematic bias. This also holds true for the elevated infection risks in essential workers, which is actually corroborated by a large body of research (9–11).

Regarding differential likelihood of testing, van Tongeren et al state that “[i]t is relatively simple to address this problem by using a test-negative design” (1). As van Tongeren et al describe, this is a case–control approach only including individuals who were tested (without considering those who were not tested). However, the proposed analytical strategy can lead to another (more serious) selection bias if testing proportions and/or testing criteria differ between groups (12). This can be easily illustrated when comparing the results based on a time-incidence design with those obtained by a test-negative design as shown in table 1.

**Table 1:** Comparison of two analytical approaches to estimate the association between occupation and SARS-CoV-2 infection during the first wave of the COVID-19 pandemic in Germany (1 February–31 August 2020). Data from the German National Cohort (NAKO). [OR=odds ratios; IRR=incidence rate ratios; CI=confidence interval; KldB 2010 = Klassifikation der Berufe 2010 (German Classification of Occupations 2010)]

	Time-incidence design^1^(N=108 960)		Test-negative design^2^(N=6 064)	
	
	IRR^3^	95% CI	OR^3^	95% CI
Essential occupations				
Non-essential workers	1.00		1.00	
Essential workers	1.96	1.59-2.41	0.88	0.71–1.09
Skill level (5^th^ digit KldB 2010)				
Unskilled or semi-skilled	1.21	0.67-2.20	1.21	0.62–2.36
Skilled activities	1.00		1.00	
Complex activities	1.15	0.87-1.52	1.13	0.84–1.51
Highly complex activities	1.39	1.08-1.80	1.14	0.87–1.48
Supervisory/managerial responsibility (4^th^ digit KldB 2010)				
No	1.00		1.00	
Supervisor	0.72	0.36-1.47	0.83	0.40–1.73
Manager	1.51	1.01-2.24	1.43	0.93–2.18

1 The time-incidence design uses all workers (tested or not tested).

2 The test-negative design uses only workers who were at least once tested for SARS-CoV-2.

3 OR based on logistic regression analysis. IRR based on robust Poisson regression with person-days at risk defined as exposure variable. Estimates were adjusted for age group (in five-year increments), sex, migration background, study centre, weekly working hours, and self-employment, and skill-level and supervisory/managerial responsibility (for the variable essential occupations) or for occupational segment (1^st^-2^nd^ digit of the KldB-2010) (for the variables skill level and supervisory/managerial responsibility). Separate models were calculated for each indicator.

Both approaches show similar results in terms of vertical occupational differences. Infection was more common if individuals had a high skill level or had a managerial position, but associations were stronger in the time-incidence design and did not reach statistical significance in the test-negative design (as indicated by the confidence intervals overlapping “1”). Unfortunately, the test-negative approach relies on a strongly reduced sample size and thus results in greater statistical uncertainty and loss of statistical power (13). In contrast, the test-negative design yields a different picture when estimating the association between essential occupation and infection risk: In this analysis, essential workers did not differ from non-essential workers in their chance of being infected with SARS-CoV-2 (the test-negative design even exhibits a lower chance for essential workers). This is rather counter-intuitive and is not in accordance with what we know about the occupational hazards of healthcare workers during the pandemic (14). The main problem is that proportions of positive tests are highly unreliable when testing proportions and/or testing criteria differ between groups. As essential workers were tested more often without being symptomatic (due to employer requirements), a lower proportion of positive tests in this group does not necessarily correspond to a lower risk of infection.

Consequently, we are not convinced that the test-negative design should be the ‘gold standard’ for studying risk factors for SARS-CoV-2 infections (15). Especially problematic is the loss of statistical power (increasing the probability of a type II error) and the low validity of the test-positivity when test criteria and/or test proportions differ between groups.

## References

[1] van Tongeren M, Rhodes S, Pearce N (2022). Occupation and SARS-CoV-2 infection risk among workers during the first pandemic wave in Germany:potential for bias. Scand J Work Environ Health.

[2] Reuter M, Rigó M, Formazin M, Liebers F, Latza U, Castell S (2022). Occupation and SARS-CoV-2 infection risk among 108 960 workers during the first pandemic wave in Germany. Scand J Work Environ Health.

[3] Busse R, Blümel M, Knieps F, Bärnighausen T (2017). Statutory health insurance in Germany:a health system shaped by 135 years of solidarity, self-governance, and competition. Lancet.

[4] Wachtler B, Michalski N, Nowossadeck E, Diercke M, Wahrendorf M, Santos-Hövener C (2020). Socioeconomic inequalities in the risk of SARS-CoV-2 infection –First results from an analysis of surveillance data from Germany. J Heal Monit.

[5] Plümper T, Neumayer E (2020). The pandemic predominantly hits poor neighbourhoods?SARS-CoV-2 infections and COVID-19 fatalities in German districts. Eur J Public Health.

[6] Abedi V, Olulana O, Avula V, Chaudhary D, Khan A, Shahjouei S (2021). Racial, Economic, and Health Inequality and COVID-19 Infection in the United States. J Racial Ethn Heal Disparities.

[7] Mukherji N (2022). The Social and Economic Factors Underlying the Incidence of COVID-19 Cases and Deaths in US Counties During the Initial Outbreak Phase. Rev Reg Stud.

[8] Beese F, Waldhauer J, Wollgast L, Pförtner T, Wahrendorf M, Haller S (2022). Temporal Dynamics of Socioeconomic Inequalities in COVID-19 Outcomes Over the Course of the Pandemic—A Scoping Review. Int J Public Health.

[9] Nguyen LH, Drew DA, Graham MS, Joshi AD, Guo C-G, Ma W (2020). Risk of COVID-19 among front-line health-care workers and the general community:a prospective cohort study. Lancet Public Heal.

[10] Chou R, Dana T, Buckley DI, Selph S, Fu R, Totten AM (2020). Epidemiology of and Risk Factors for Coronavirus Infection in Health Care Workers. Ann Intern Med.

[11] Stringhini S, Zaballa M-E, Pullen N, de Mestral C, Perez-Saez J, Dumont R (2021). Large variation in anti-SARS-CoV-2 antibody prevalence among essential workers in Geneva, Switzerland. Nat Commun.

[12] Accorsi EK, Qiu X, Rumpler E, Kennedy-Shaffer L, Kahn R, Joshi K (2021). How to detect and reduce potential sources of biases in studies of SARS-CoV-2 and COVID-19. Eur J Epidemiol.

[13] Cohen J (2013). Statistical Power Analysis for the Behavioral Sciences.

[14] The Lancet (2020). The plight of essential workers during the COVID-19 pandemic. Lancet.

[15] Vandenbroucke JP, Brickley EB, Pearce N, Vandenbroucke-Grauls CMJE (2022). The Evolving Usefulness of the Test-negative Design in Studying Risk Factors for COVID-19. Epidemiology.

